# Chronic Distal Biceps Tendon Tear Reconstruction With Tibialis Anterior Allograft

**DOI:** 10.1016/j.eats.2021.04.017

**Published:** 2021-07-13

**Authors:** Raffy Mirzayan, Emily S. Mills

**Affiliations:** a Department of Orthopaedics, 1. Kaiser Permanente Southern California, Baldwin Park, California, U.S.A.; b Department of Orthopaedics, University of Southern California Keck School of Medicine, Los Angeles, California, U.S.A.

## Abstract

Chronic distal biceps tendon ruptures present a unique surgical challenge due to tendon retraction and shortening as well as muscle atrophy. Several graft choices and fixation methods have been described, with no one technique proving superior to date. We describe a technique wherein a tibialis anterior tendon allograft is Pulver-Taft weaved through the muscle belly to achieve incorporation with the muscle and tendonous fascia to achieve superior pull-out strength.

Distal biceps tendon ruptures occur at a rate of 2.5 per 100,000 and result from eccentric overloading of the elbow in flexion.^1^ Patients may present with a characteristic reverse Popeye deformity, a positive hook test, as well as weakness in supination and flexion.^2^^,^^3^ Acute operative repair is recommended in young, healthy patients.^4^ Patients who do not undergo acute operative repair are classified as having chronic distal biceps ruptures, which are usually defined as those greater than 4 weeks from injury.^5^ A subset of patients with chronic tears do well with continued nonoperative management; however, there are patients who have continued weakness and pain.^1^ These patients present a unique challenge. The biceps tendon is shortened, atrophied, often times “cocooned,” making primary repair impossible.^6^ Therefore, the only surgical option for these patients is to undergo distal biceps tendon reconstruction.

Several techniques have been described for distal biceps tendon reconstruction. The use of semitendinosus, gracilis, tibialis anterior, Achilles, flexor carpi radialis, and lacertus fibrosis grafts have all been described.^7^, ^8^, ^9^, ^10^, ^11^, ^12^ Several techniques for proximal graft attachment were described in these studies, including end-to-end, Pulver-Taft, and onlay methods. A recent review and meta-analysis of these repairs showed no difference in complications between autograft and allograft tendon reconstruction.^13^ However, the authors concluded that there is still no consensus on which graft and insertion technique is best. Here, we present a technique using a tibialis anterior allograft and Pulver-Taft implantation method.

## Surgical Technique (With Video Illustration)

**Fig 1:**
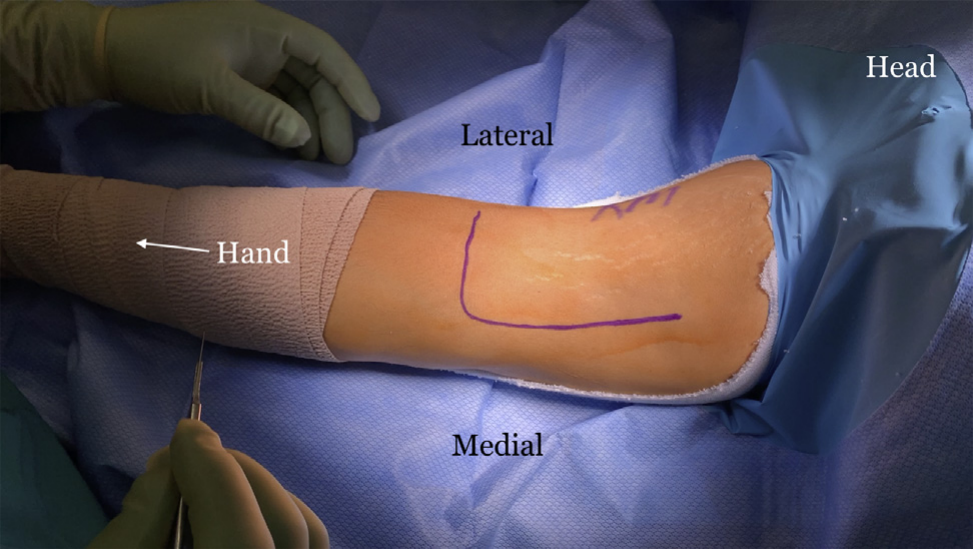
The right arm of the patient is seen in the supine position. A curvilinear incision is used.

**Fig 2:**
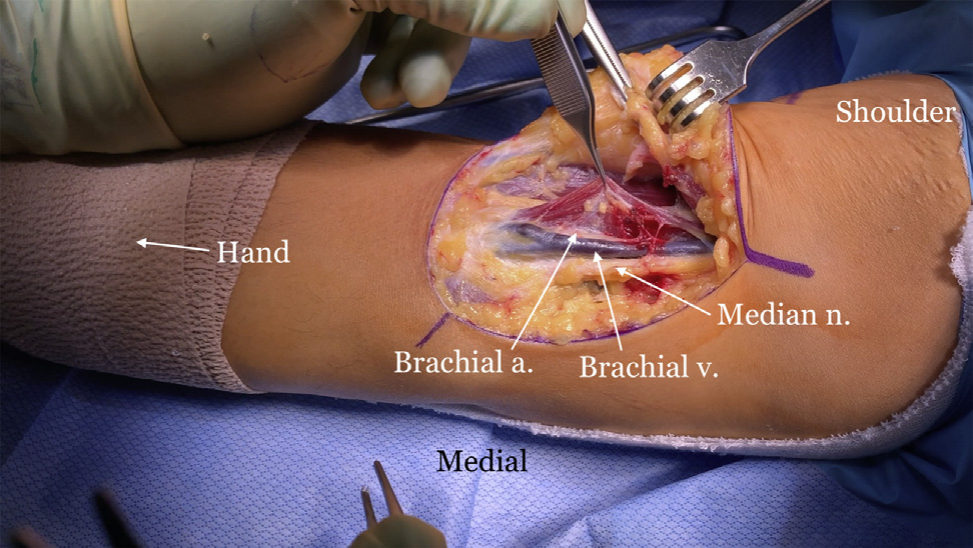
The right arm of the patient is seen in the supine position. The dissection is carried out from medial to lateral. The medial antebrachial cutaneous nerve (not seen here) is dissected first, followed by the median nerve, brachial vein, and artery.

**Fig 3:**
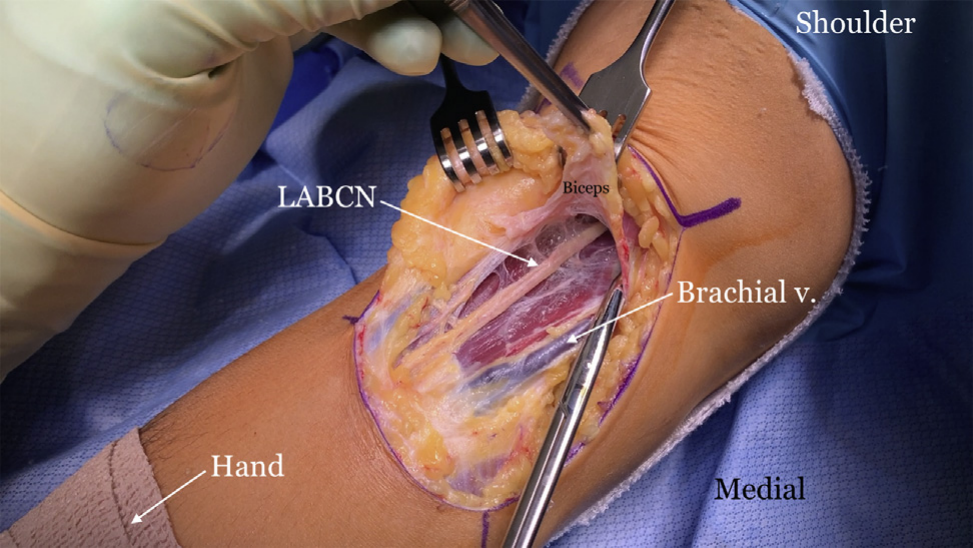
The right arm of the patient is seen in the supine position. The lateral antebrachial cutaneous nerve (LABCN) is identified between the biceps and brachialis muscles.

**Fig 4:**
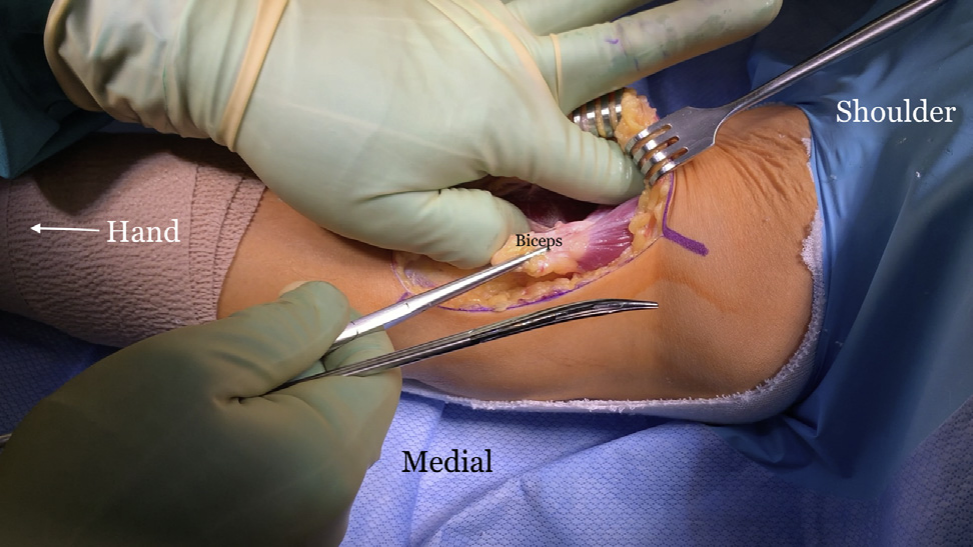
The right arm of the patient is seen in the supine position. Using the surgeon’s finger, all adhesions are freed up 360° around the biceps muscle belly to mobilize the muscle.

**Fig 5:**
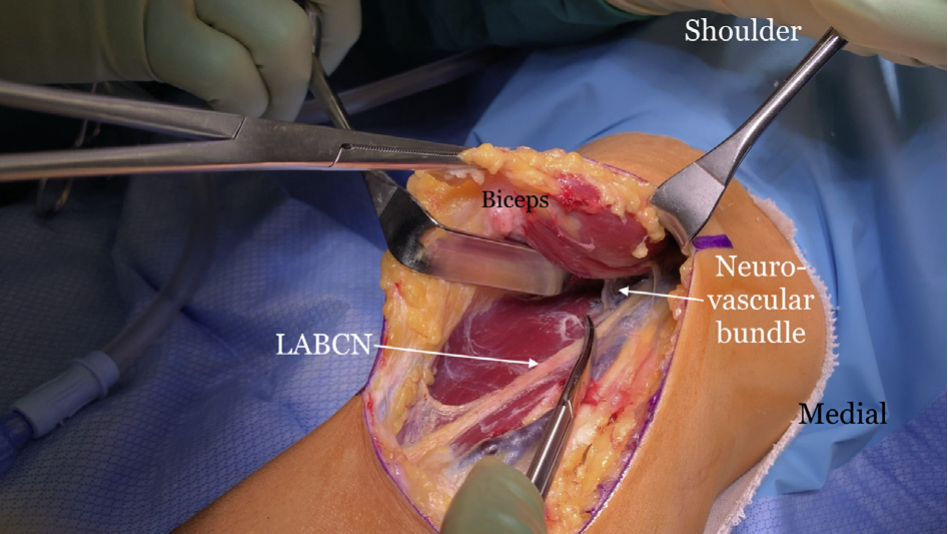
The right arm of the patient is seen in the supine position. A neurovascular bundle can be seen entering the biceps muscle from underneath and should not be damaged. (LABCN, lateral antebrachial cutaneous nerve.)

**Fig 6:**
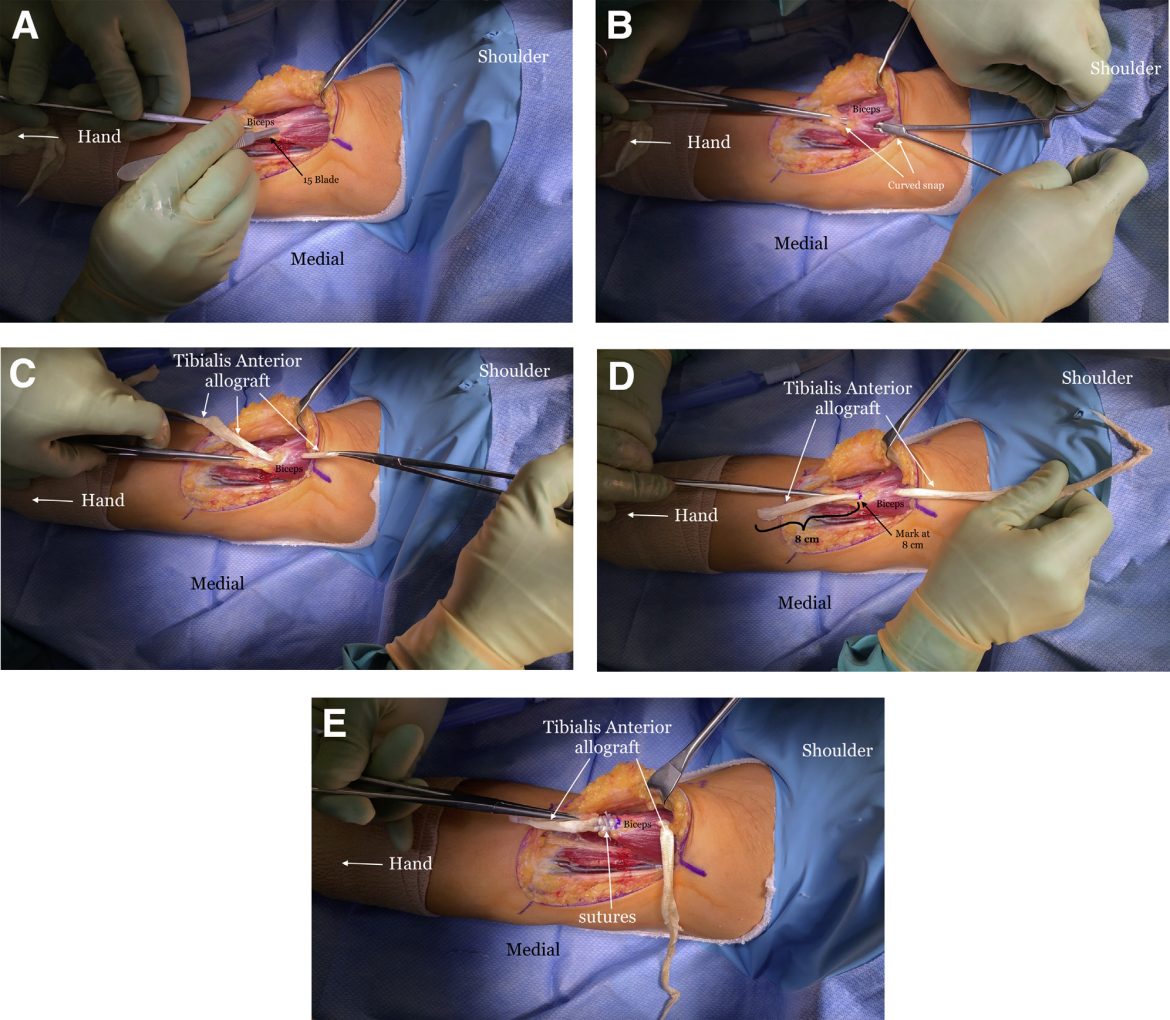
The right arm of the patient is seen in the supine position. (A) A small incision is made with a #15 blade at the musculotendinous junction. (B) A curved snap is passed from the muscle belly exiting through the incision. (C) The thin end of the tibialis anterior allograft is then grasped by the snap and passed through the incision and brought out the muscle belly. (D) Approximately 8 cm of allograft tendon is left (marked) at the musculotendinous junction. (E) the allograft tendon is then sutured to the native biceps tendon stump with SutureTape (Arthrex, Naples, FL).

The indications and contraindications are listed in Table 1, and the technique is demonstrated in Video 1. The patient is positioned in the supine position with a standard hand table attachment under general anesthesia. The procedure is performed under tourniquet. A curvilinear (“L-shaped”) incision is made starting at the elbow flexion crease in the anticubital fossa and carried medially over the intermuscular septum (Fig 1). Blunt dissection is performed in a medial-to-lateral direction of the brachium. The medial antebrachial cutaneous nerve is identified first but occasionally is not seen, as in this particular case. The median nerve and the brachial artery and vein are the next structures to be identified and dissected (Fig 2). The medial aspect of the biceps muscle and tendon are often scarred to the neurovascular bundle and need to be freed to mobilize the biceps muscle. The lateral antebrachial cutaneous nerve, sandwiched between the biceps and brachialis, is identified next (Fig 3). The stump of the biceps tendon is then grasped and pulled so the muscle belly can be released 360° circumferentially from surrounding tissue to increase mobility and excursion (Fig 4). Care should be taken when dissecting posteriorly underneath the muscle, as neurovascular bundles that enter the biceps can be encountered and should not be disrupted (Fig 5). A tibialis anterior allograft (LifeNet Health, Virginia Beach, VA) is used. We recommend selecting the longest possible graft (with a minimum length of 270 mm but ideally more than 300 mm) and a folded diameter of 9.5 or 10 mm. The tendonous portion of the allograft, which is thick and round, is used as the insertion to the radial tuberosity. The other end is the thinner and broad and is woven into the muscle belly in a Pulver-Taft weave. A #15 blade is then used to create a stab incision at the distal end of the biceps muscle, just proximal to the tendon stump, through the muscle belly (Fig 6A). A curved snap is used as a shuttle to pass the graft though this passage (Fig 6 B and C). The allograft is pulled proximally until 8 cm of tendon is left at the musculotendinous junction (Fig 6D). The allograft is then sutured to the native, shortened tendon stump (Fig 6E). The proximal end of the graft is then weaved though the proximal muscle belly in a Pulver-Taft fashion.

**Table 1:** Indications and Contraindications

Indications
1. Chronic distal biceps rupture where tendon cannot be primarily repaired
2. Elbow flexion and supination weakness
3. Failed nonoperative management
Contraindications
1. Chronic tear where native tendon can be directly fixed to the tuberosity
2. Active infection
3. Patients without any symptoms related to the biceps brachii

**Fig 7:**
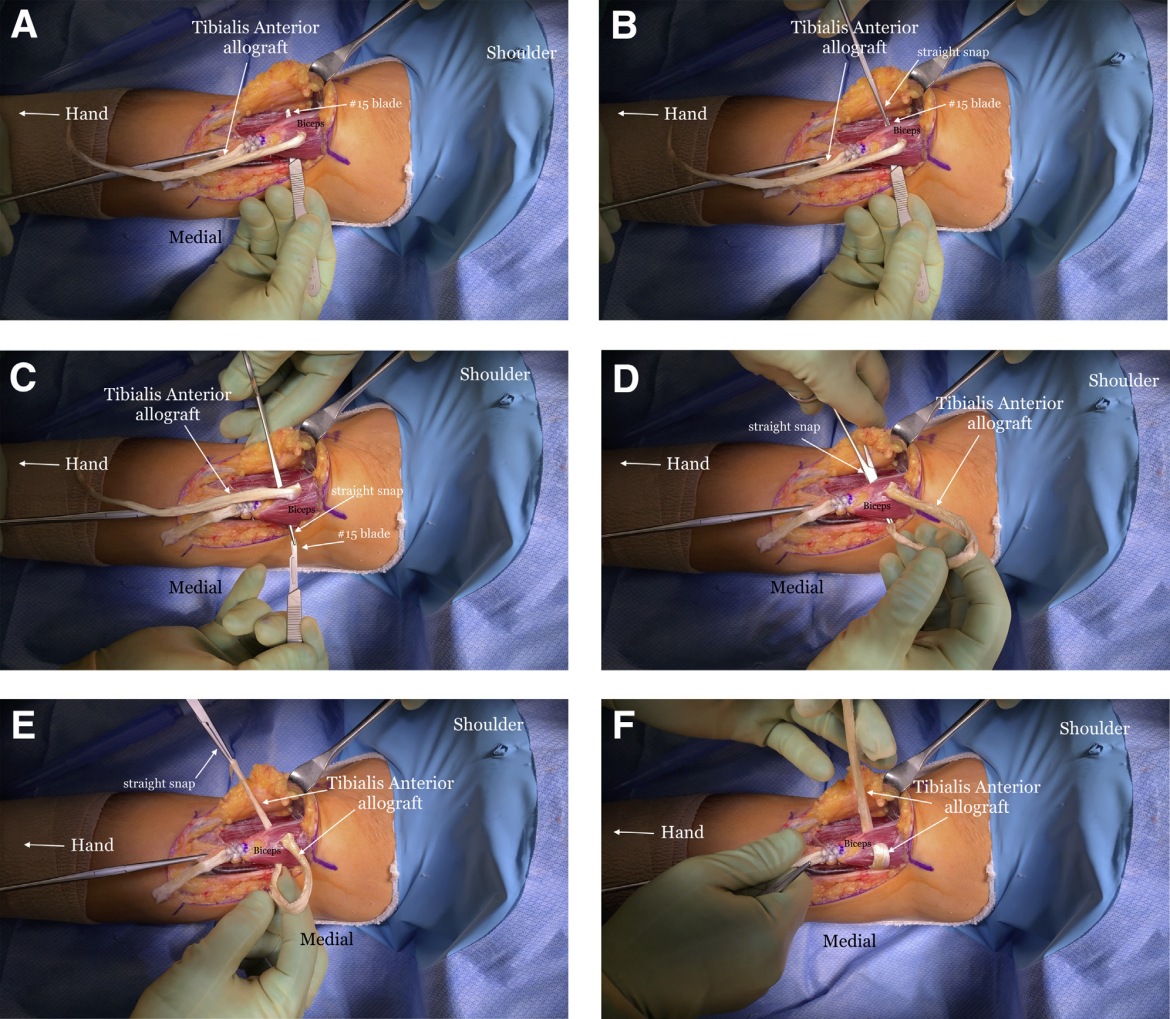
The right arm of the patient is seen in the supine position. (A) A #15 blade is passed through the muscle belly in a medial to lateral direction. (B) A straight snap grasps the #15 blade. (C) The #15 blade is pulled out of the muscle with the straight snap attached to it. (D) The allograft tendon is placed into the snap. (E) The allograft tendon is pulled through the muscle belly. (F) The allograft tendon is tightened around the muscle belly.

**Fig 8:**
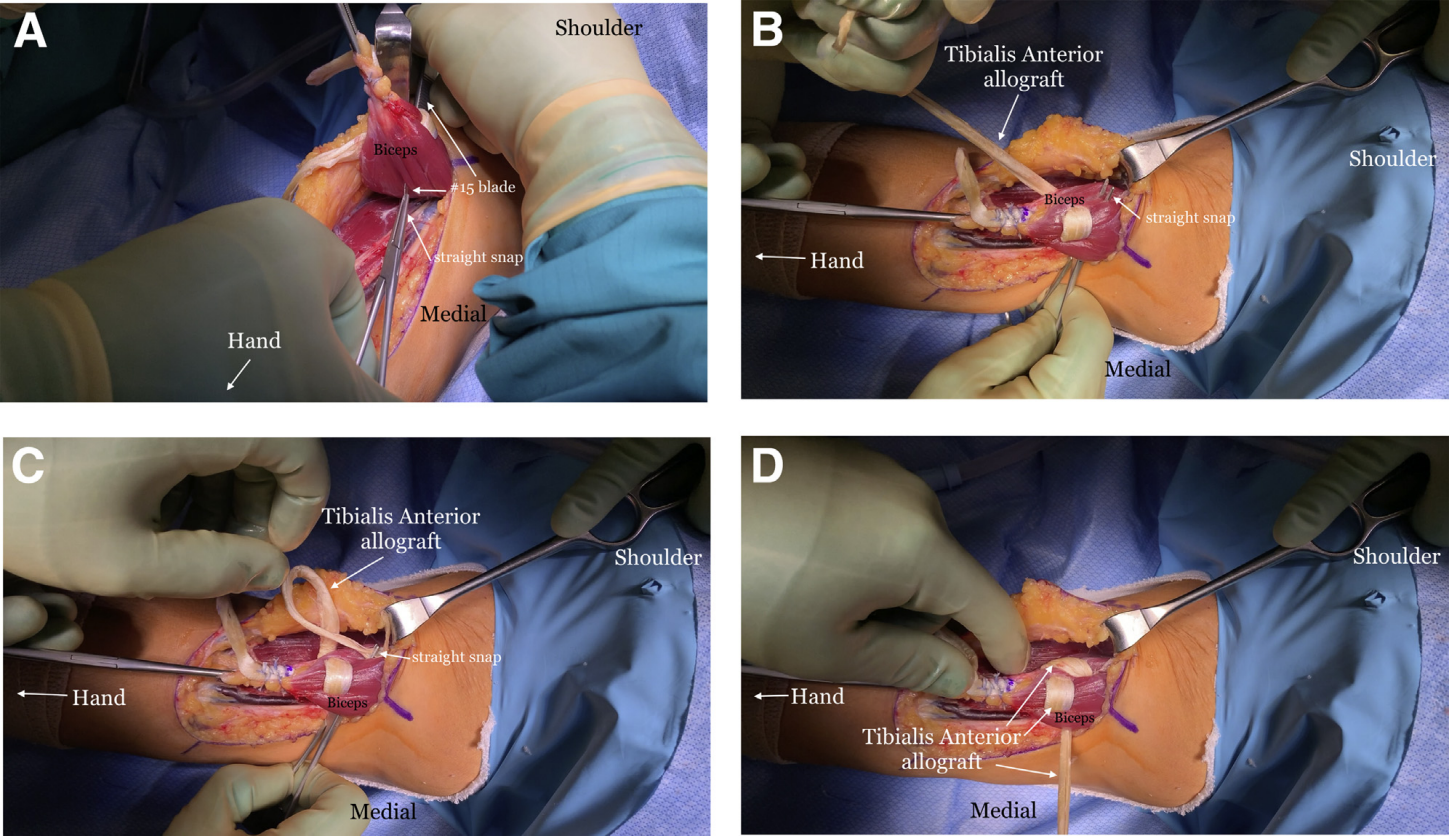
The right arm of the patient is seen in the supine position. (A) A #15 blade is passed through the muscle belly in an anterior to posterior direction and the straight snap grasps the blade. (B) The straight snap is passed through the muscle belly. (C) The thin end of the allograft tendon is grasped by the snap. (D) The allograft tendon is then passed through the muscle belly in an anterior to posterior direction.

**Fig 9:**
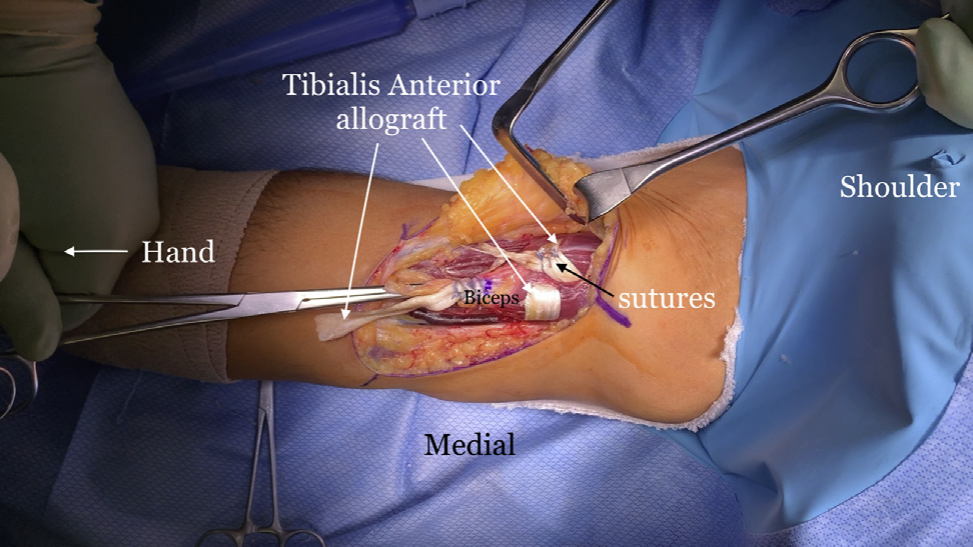
The right arm of the patient is seen in the supine position. The allograft tendon is then sutured to itself in multiple places with SutureTape (Arthrex, Naples, FL).

**Fig 10:**
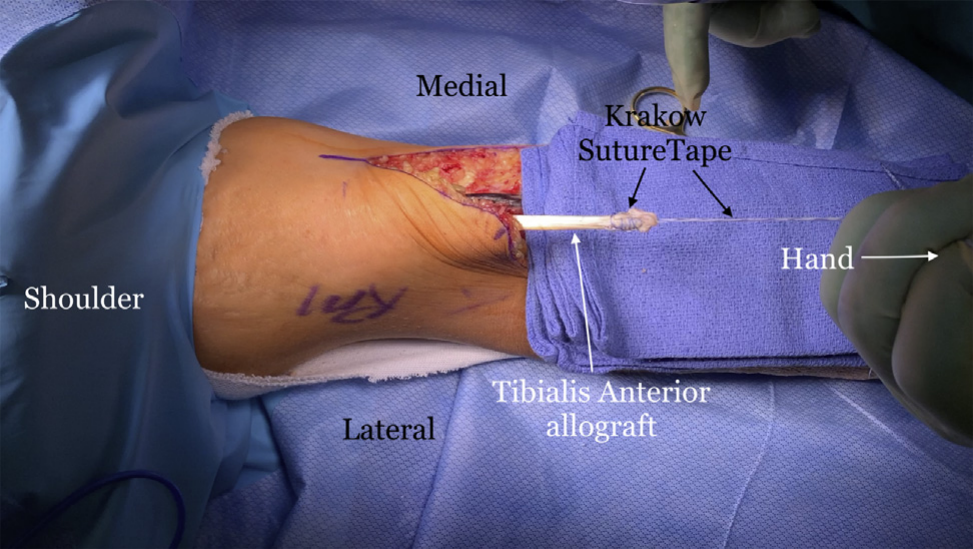
The right arm of the patient is seen in the supine position. The end of the allograft tendon is then sutured in a Krakow fashion with SutureTape (Arthrex, Naples, FL).

A #15 blade is used to create passages through the muscle, starting from medial to lateral, just proximal to the insertion site of the allograft (Fig 7A). A straight snap is placed on the end of the #15 blade once the blade is through the muscle (Fig 7B), and then they are both retracted through the muscle (Fig 7C). The proximal end of the graft is grasped using the straight snap and shuttled though the muscle (Fig 7 D-F). The graft is then passed through the muscle belly in an anterior-to-posterior direction (Fig 8). The tendon graft is passed as many times as its length allows. SutureTape (Arthrex, Naples, FL) is then used to suture the proximal part of the graft to itself (Fig 9). A SutureTape is sewn approximately 20-25 mm up the tendinous end of the graft in a Krakow fashion (Fig 10).

**Fig 11:**
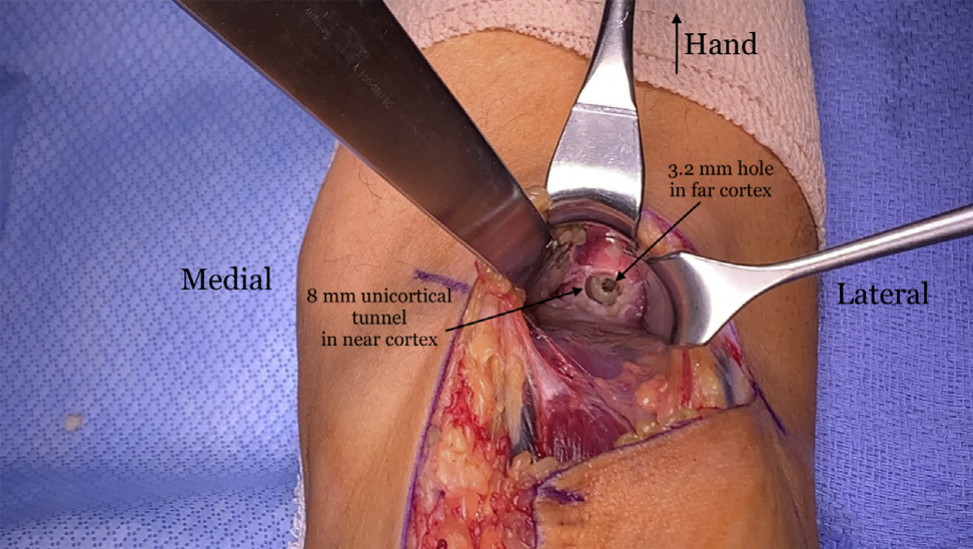
The right arm of the patient is seen in the supine position. A unicortical 8mm diameter tunnel is reamed in the radial tuberosity over a 3.2-mm spade-tipped guide pin that was placed bicortically.

**Fig 12:**
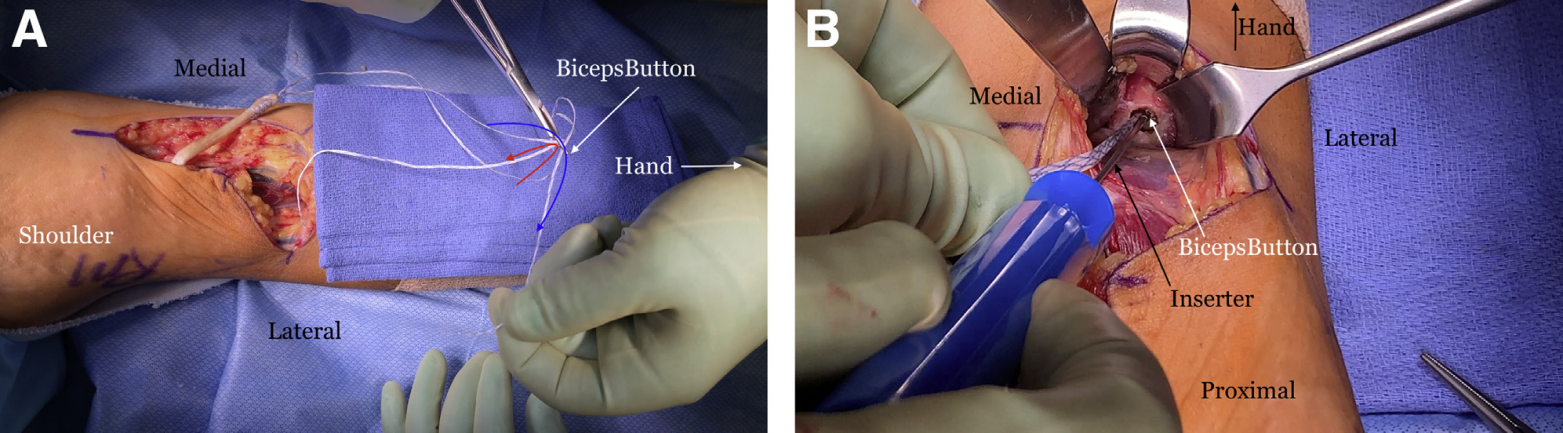
The right arm of the patient is seen in the supine position. (A) The ends of the SutureTape (Arthrex, Naples, FL) are then passed through a BicepsButton (Arthrex, Naples, FL) in opposite directions in order to allow the button to flip easier on the far cortex. (B) An inserter is then used to pass the button through the tunnel and out of the 3.2 mm hole in the far cortex.

The radial tuberosity is dissected out and identified by digital probing and by pronating and supinating the forearm. If there is any question of where the radial tuberosity is, fluoroscopy may be used to ensure proper placement. Gentle retraction is used to avoid excessive retraction of the posterior interosseous nerve. The graft tendon diameter is measured and is usually between 6 or 7 mm. A reamer one size up is selected. For instance, for a graft size of 7 mm, an 8-mm low profile reamer is used. A 3.2-mm spade-tipped guide pin is inserted bicortically in the center of the radial tuberosity. The cannulated reamer is placed over the guide pin and a unicortical tunnel is created (Fig 11). Copious irrigation is used to remove bone reamings. The previously placed SutureTape in the distal end of the graft is passed through a BicepsButton (Arthrex) (Fig 12A), and the button is placed through the tunnel and then flipped on the far cortex of the radius (Fig 12B).

**Fig 13:**
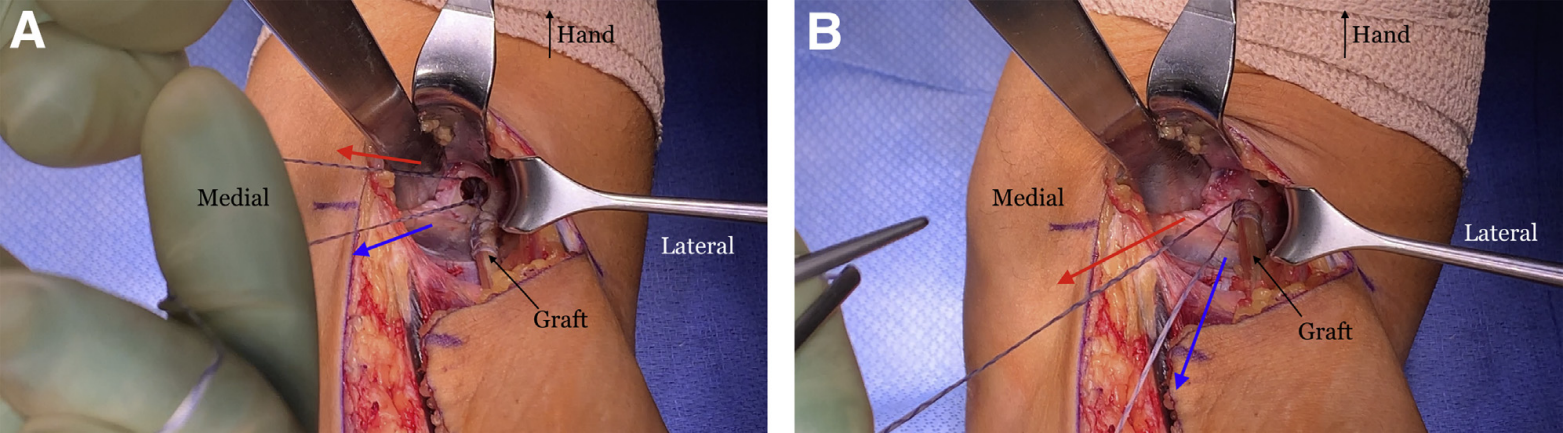
The right arm of the patient is seen in the supine position. (A) Once the button is flipped on the far cortex, the ends of the SutureTape (Arthrex, Naples, FL) are pulled sequentially in a tension slide manner to reduce the allograft tendon into the tunnel. (B) The allograft tendon is seen reduced into the tunnel.

**Fig 14:**
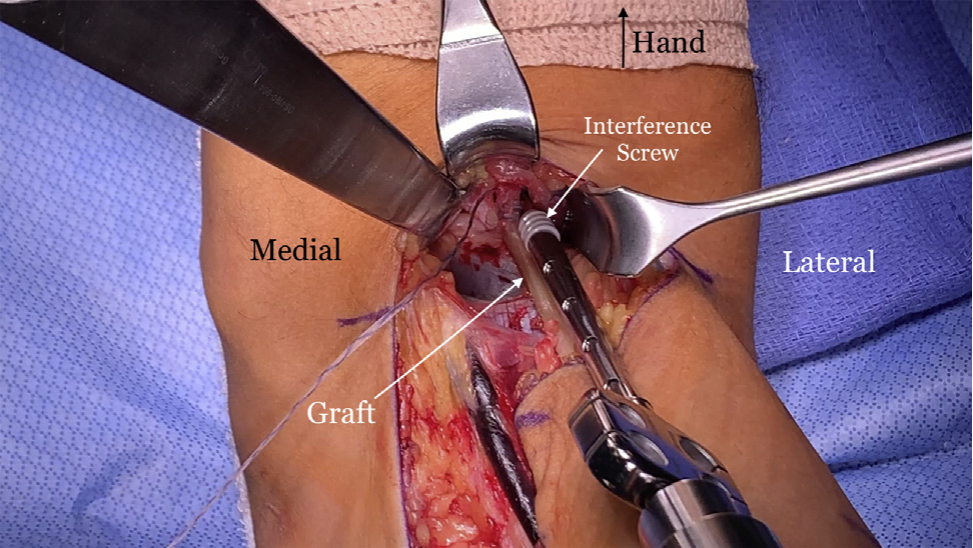
The right arm of the patient is seen in the supine position. A 7 × 10-mm BioComposite interference screw (Arthrex, Naples, FL) is then placed on the lateral side of the tunnel, pushing the allograft tendon in a more ulnar and anatomic position.

Next, the suture tails are pulled in a tension slide technique and the tendon is reduced into the tunnel (Fig 13). The sutures are then tied, and a knot pusher is used to push the knots into the tunnel as close to the bottom of the tunnel as possible. We tension the graft with the arm in approximately 40° of flexion. A 7 × 10-mm BioComposite or PEEK screw (Arthrex) is placed into the radial side of the tunnel, pushing the tendon graft in an ulnar direction to recreate the native distal biceps insertion (Fig 14). The tourniquet is released and any bleeding identified and controlled. The surgical site is irrigated thoroughly. The incision is closed with 3-0 monofilament suture subcutaneously. Adhesive tape strips are applied, followed by a sterile dressing. A well-padded posterior molded splint is placed with the elbow at 90° of flexion and the forearm in neutral rotation, with the wrist left free. The arm is placed in a sling. After a postoperative neurovascular check, a nerve block can be placed by the anesthesia team for patient comfort if desired.

## Postoperative Management

The patient’s arm is placed in a long arm posterior molded splint with the elbow at 90°, neutral forearm rotation, and the wrist free, until the first postoperative visit at 7 to 10 days. Between the initial visit until the 2 months postoperatively, active and active assist range of motion is performed to regain full range of motion. Between 2 and 4 months, patients work on resisted exercises with bands. From 4 to 6 months, they start strength training and are allowed to increase as tolerated and are cleared between 5 and 6 months to return without restrictions.

## Discussion

Chronic distal biceps tendon ruptures are a difficult problem to treat due to tendon shortening and retraction. Several techniques and graft choices have been described, with no method found to be superior to others to date. A recent meta-analysis by Litowski et al.^13^ compared autografts and allografts as well as graft fixation technique and found no difference in postoperative range of motion, strength, patient-reported outcomes, or re-rupture rates. The autograft cohort had increased complications due to graft-site morbidity. There remain no guidelines on which graft or implantation technique is best.

Cross et al.^9^ published a case series of patients who underwent reconstruction using a tibialis anterior graft with an end-to-end implantation technique. They found that early results for their cohort of patients were good to excellent. Snir et al.^8^ studied several different allograft types, including one patient who underwent repair with tibialis anterior tendon secured with an onlay technique. In this technique, the musculotendinous end of the graft is laid on top of the muscle belly and secured with suture. The authors concluded that this method was safe an effective for chronic distal biceps tendon repair, although their preference in this study was Achilles tendon allograft.

This article describes a novel surgical technique using tibialis anterior tendon allograft that is weaved through the muscle belly of the biceps in a Pulver-Taft method for to achieve immediate secure implantation of the tendon into the muscle in patients with chronic distal biceps tendon ruptures. The indications are listed in Table 1. The risks and limitations of this procedure are minimal (Table 2), and pearls and pitfalls are outlined in Table 3. There is a potential risk of disease transmission form an allograft. We prefer grafts over a length of 320 mm, and this is not always available. We believe that the Pulver-Taft technique for implantation provides improved pull out strength of the graft as compared to the onlay technique, although this has not been shown in any studies to date. Further clinical research is necessary to study both graft selection and implantation method.

**Table 2:** Advantages and Disadvantages

Advantages
1. Allograft avoids donor-site morbidity
2. Pulver-Taft method allows for integration of graft into native biceps muscle belly
Risks and Limitations
1. Cost of allograft
2. Implant costs
3. Posterior interosseous nerve at risk of palsy especially if a tipped retractor (Hohmann) retractor is used on the lateral side of the radial tuberosity
4. Limitation—can take up to 6 months to fully recover and be released from care

**Table 3:** Pearls and Pitfalls

Pearls
1. Use the longest graft available, preferably 300 mm or longer
2. Use fluoroscopy to help locate the radial tuberosity if necessary
3. Suture graft to itself after weaving through muscle belly
4. Place interference screw on the radial side of the tunnel to medialize the graft
Pitfalls
1. Use gentle retraction when reaming the radial insertion site to avoid posterior interosseous nerve palsy
2. Avoid excessive retraction of lateral antebrachial cutaneous nerve, as this is a common postoperative nerve palsy^13^
3. If a short graft is used, may not be able to make several passes through the muscle belly

## Supplementary Data

Video 1 The patient is placed in the supine position, and a nonsterile tourniquet is used. An L-shaped curvilinear incision is used starting in the antecubital crease and carried medially along the medial intermuscular septum. The dissection is carried out in a medial-to-lateral direction. The medial antebrachial cutaneous nerve should be identified first but is not seen in this particular case. The neurovascular bundle is often scarred to the biceps tendon stump and muscle and should be dissected out, followed by the lateral antebrachial cutaneous nerve, which is found between the biceps and brachialis. This nerve is often encased in scar tissue and can take some time to dissect out. The biceps muscle belly is then circumferentially freed from any adhesions in order to mobilize the muscle. Digital dissection can be carried out instead of sharp dissection. A small incision is made with a #15 blade at the musculocutaneous junction. A curved snap is passed from the muscle belly exiting through this incision. The thin end of the tibialis anterior allograft is then grasped by the snap and passed through the incision and brought out of the muscle belly. Approximately 8 cm of allograft tendon is left at the musculotendinous junction. The allograft tendon is then sutured to the native biceps tendon stump. A #15 blade is passed through the muscle belly in a medial to lateral direction. A straight snap grasps the #15 blade. The #15 blade is pulled out of the muscle with the straight snap attached to it. The allograft tendon is placed into the snap and pulled through the muscle belly. A #15 blade is then passed through the muscle belly in an anterior to posterior direction and the straight snap grasps the blade which is passed through the muscle belly. The thin end of the allograft tendon is grasped by the snap and passed through the muscle belly in an anterior to posterior direction. These steps are then repeated as many times as possible to pass as much graft material through the muscle belly as allowed by the length of the allograft. The allograft limbs are then sutured to themselves at multiple locations to secure the graft into the muscle belly. To identify the radial tuberosity, the surgeon’s finger is placed medial to the lateral antebrachial cutaneous nerve and on top of the brachialis muscle and advanced into the forearm. The digit is aimed laterally toward the radius. By pronating and supinating the forearm, the rough ridge of the radial tuberosity can be palpated with the finger. A Hohmann retractor is placed medially and a Richardson retractor placed laterally and distally. The end of the tendon is then sutured in a Krakow fashion with SutureTape (Arthrex, Naples, FL). Approximately 5 throws on each side are adequate, usually at a length of approximately 20-25 mm up the tendon. The end of the tendon is then trimmed and tubularized to allow easy passage into the tunnel. The diameter of the sutured graft end is then determined. In this case it was 7mm. A 3.2-mm spade-tipped guide pin is then placed bicortically in the center of the radial tuberosity. In this case, an 8-mm low-profile reamer is used to create a unicortical tunnel. This generates bone shavings that need to be irrigated and suctioned out of the wound to avoid heterotopic ossification formation. The ends of the SutureTape that was used to suture the graft end are then passed through the biceps button in opposite directions. This makes it easier for the button to flip on the far cortex. The button is placed onto an inserter, which advances is it through the tunnel and out of the 3.2-mm hole in the far cortex. Once the button is released, all 4 SutureTape limbs are pulled to flip the button and engage it against the cortex. The limbs of the SutureTape are then pulled sequentially in a tension slide manner, reducing the tendon into the tunnel. An arthroscopic knot pusher is then used to tie and advance half hitch knots to the bottom of the tunnel. This secures the tendon graft in the tunnel and prevents it from backing out of the tunnel. The longer of the 2 suture limbs is then placed through the canulated tenodesis driver, which is used to place a 7 × 10-mm biocomposite interference screw in the tunnel. Care should be taken to place the screw on the lateral side of the tunnel so the tendon is pushed to a medial and more anatomic position. Knots are then tied with the SutureTapes on top of the screw for additional fixation and to avoid the screw from backing out of the tunnel. The repair is performed typically with the elbow in 30 to 40° of flexion to assure the biceps is properly tensioned.

ICMJE author disclosure forms
